# Driving following Kava Use and Road Traffic Injuries: A Population-Based Case-Control Study in Fiji (TRIP 14)

**DOI:** 10.1371/journal.pone.0149719

**Published:** 2016-03-01

**Authors:** Iris Wainiqolo, Berlin Kafoa, Bridget Kool, Elizabeth Robinson, Josephine Herman, Eddie McCaig, Shanthi Ameratunga

**Affiliations:** 1 School of Population Health, University of Auckland, Auckland, New Zealand; 2 College of Medicine, Nursing and Health Sciences, Fiji National University, Suva, Fiji; Beihang University, CHINA

## Abstract

**Objective:**

To investigate the association between kava use and the risk of four-wheeled motor vehicle crashes in Fiji. Kava is a traditional beverage commonly consumed in many Pacific Island Countries. Herbal anxiolytics containing smaller doses of kava are more widely available.

**Methods:**

Data for this population-based case-control study were collected from drivers of ‘case’ vehicles involved in serious injury-involved crashes (where at least one road user was killed or admitted to hospital for 12 hours or more) and ‘control’ vehicles representative of ‘driving time’ in the study base. Structured interviewer administered questionnaires collected self-reported participant data on demographic characteristics and a range of risk factors including kava use and potential confounders. Unconditional logistic regression models estimated odds ratios relating to the association between kava use and injury-involved crash risk.

**Findings:**

Overall, 23% and 4% of drivers of case and control vehicles, respectively, reported consuming kava in the 12 hours prior to the crash or road survey. After controlling for assessed confounders, driving following kava use was associated with a four-fold increase in the odds of crash involvement (Odds ratio: 4.70; 95% CI: 1.90–11.63). The related population attributable risk was 18.37% (95% CI: 13.77–22.72). Acknowledging limited statistical power, we did not find a significant interaction in this association with concurrent alcohol use.

**Conclusion:**

In this study conducted in a setting where recreational kava consumption is common, driving following the use of kava was associated with a significant excess of serious-injury involved road crashes. The precautionary principle would suggest road safety strategies should explicitly recommend avoiding driving following kava use, particularly in communities where recreational use is common.

## Introduction

Of the approximately 1.24 million road deaths each year, almost 95% occur in low and middle income countries [1]. Furthermore, the burden of road traffic injuries in many of these countries is projected to increase sharply in coming decades imposing substantial demands on poorly resourced healthcare systems, compromising the development of fragile economies, and making millions of people vulnerable to increased poverty and on-going suffering [1].

These inequitably borne consequences highlight the importance of implementing effective road safety strategies that take particular account of risk factors in less-resourced settings [1–3]. We were concerned that while alcohol, some medicines and drugs (licit and illicit) [1, 4–7] are well-recognised risk factors in high-income countries, little is known of the possible contribution to crashes from kava, a traditional soporific substance commonly consumed in several small island developing states in the Pacific region.

Kava is a popular intoxicating beverage extracted from the roots of the plant *Piper methysticum*, a perennial shrub native to most Pacific Island countries and territories [8]. As noted in our review of the published literature[9], while some authorities recommend caution when driving and operating heavy machinery following the use of high doses of kava, there is a notable lack of epidemiological studies investigating the contribution of the recreational use of kava to road traffic injuries. The published literature on the potential risks involved are confined to experimental studies on the effects of small doses of kava when operating driving simulators, the results of which are inconclusive [9]. These doses (e.g., 180–300 mg kavalactones) are consistent with the concentrations of kava found in dietary supplements that are becoming more widely available in Western countries as herbal remedies for the relief of anxiety, menopausal symptoms and other putative health benefits [10–12].

Recreational use of kava in many Pacific Island countries typically involves doses more than 50 times that in herbal and therapeutic preparations [13]. We investigated the association between driving after consuming kava and serious injury-involved four-wheeled motor vehicle crashes in Fiji.

## Materials and Methods

### Study setting and design

A population-based case-control study was undertaken in Viti Levu, Fiji, from July 1, 2005 to December 31, 2006. Viti Levu is home to 70% of Fiji’s resident population of 837,271 [14]. About 20% of the public roads on the island are tar sealed, and cars and commercial vehicles account for approximately 86% of the driving fleet [15]. This study was approved by the Fiji National Research and Ethics Review Committee (FNRERC Ref No. 023–2004), Suva, Fiji and the University of Auckland Human Participants Ethics Committee, Auckland, New Zealand (UAHPEC Ref No. 2005/095). All participants provided written informed consent as approved by the Ethics committees. The roadside survey was granted approval by the Fiji Police Force Highway Unit.

Unintentional injuries such as road traffic injuries are rare events and are therefore suited to a case-control design[16]. For the valid conduct of a case-control study, both the cases and controls need to come from the same study base [17, 18]. Drawing on the deductive research methodology employed in the Auckland Car Crash Injury Study [19], the study base for this research comprised “motor vehicle driving time” (equivalent to person-time) on public roads in Viti Levu.

Eligible vehicles included motorised four-wheeled motor vehicles such as private cars, taxis, minibuses, pick-ups, trucks, and commercial or government vehicles. Buses, motorbikes, ambulance, police vehicles, and vehicles of the Diplomatic Corp were excluded.

Due to the low risk of crashes as indicated by Fiji Police traffic data, the following were excluded from the study base: roads with an average flow of less than 200 vehicles per day and vehicles driven between the hours of 2.00am and before 5.00am [20].

### Participants

#### Case recruitment

The case and control selection process for this study has been described in detail in a previous publication arising from this research that explored the role of sleepiness in car crash injury in this population [21]. Cases comprised four-wheeled motor vehicles involved in a crash in the study base where a road user (driver, passenger or pedestrian) died or was hospitalised for 12 hours or more during the study period. For all cases (crash vehicles), the driver comprised the key informant of interest. Cases were prospectively identified from road crash-related admissions recorded at all trauma-admitting hospitals in Viti Levu. This system identified injured individuals from emergency department registers, admission records, and mortuary registers [22]. In situations where the hospitalised crash victim was someone other than the driver (e.g. a vehicle passenger or a pedestrian), the driver of the crash vehicle was identified through the assistance of Traffic Police or hospital staff and invited to participate. If the driver had died or was unable to participate due to severe injuries, proxy interviews were sought from either a passenger who was also involved in the crash or family member(s).

#### Control recruitment and sampling

Controls comprised a sample representative of motor vehicle ‘driving time’ in the study base, and were identified using a prospective two-stage cluster sample roadside survey design. Recruitment of eligible four-wheeled motor vehicles occurred at 50 randomly selected eligible road sites (longer than 400 meters and with daily traffic counts of 200 or more vehicles). This sampling approach was used to ensure controls were sampled in proportion to the amount of driving undertaken given that the exposure to risk of a crash only occurred when a person is driving. Random sampling of vehicles occurred over a two-hour period at safe sections of road at each survey site on random days of the week, time of the day, and direction of travel. We also collected traffic counts for each survey of all vehicles travelling in the same direction as the vehicles that were selected for the study. This was to enable a weighting to be assigned to controls from each site that was the inverse of the proportion of all vehicles selected as controls.

In accordance with national transport protocols and institutional review board (ethics committee) requirements in Fiji, Traffic Police managed the slowing of traffic and stopping of selected vehicles. All other research procedures including the provision of information sheets and consent forms for potential participants were undertaken by the research team.

### Study instrument and variables

Cases completed an interviewer-administered questionnaire either face-to-face in hospital, at home, or via telephone. For control drivers, interviews were conducted face-to-face on-site or in another location convenient for the driver or via telephone.

The 72-item questionnaire (S1 and S2 Questionnaires) captured information about vehicle details (e.g. type of vehicle, brand, model, year of manufacture, seatbelt fitting), circumstances of the crash/survey (e.g. vehicle speed, wet road conditions), health status (e.g. sleep apnoea, depression, prescription medication use), driving experience, socio-demographic characteristics (e.g. age, gender, ethnicity, household income) and frequency of usual alcohol, kava and recreational drug use in the previous 12 months. In addition, drivers were asked if they had consumed kava or alcohol in the 12 hours prior to the crash or survey (defined as ‘acute use’), and if so the amount they had consumed. They were also asked how frequently they had consumed kava during the previous 12 months (defined as ‘usual kava use’).

Ethnicity data used in this study was self-determined by participants. The two main ethnic groups in Fiji are the indigenous Fijians (iTaukei) and Indians, who make up 57% and 38% of Fiji’s population, respectively [14]. The decision to compare the two major ethnic groups in Fiji in this study was due to their distinct cultural practices and therefore the likely need for ethnic-specific interventions.

### Measurement of kava

While we attempted to quantify the amount of kava consumed by drivers, the variable preparations, concentrations and context in which kava was consumed and shared made it difficult to quantify and standardise the amount of kava consumed. Given the resources and technology available, this study did not undertake an objective measurement of kava.

### Statistical Analysis

We formulated an analysis plan to firstly describe the characteristics of the cases and controls. Weighted proportions were used for the control data to adjust for the cluster sampling design. Secondly, the relationship between acute kava use (previous 12 hours) and car crash injury was explored. Odds ratios (ORs) and 95% confidence intervals (CIs) were obtained using unconditional logistic regression adjusted for demographic variables and other potential confounders.

The OR is a measure of association which compares the odds of an outcome (e.g. car crash) in in those exposed (e.g. consumed kava 12 hours prior to driving) to the odds of an outcome in those unexposed.[23] This is expressed in the following manner:

$$OR=\;\frac{\left(Odds\;of\;outcome\;in\;exposed\;group\right)}{\left(Odds\;of\;outcome\;in\;unexposed\;group\right)}$$

Finally, multivariable modelling was undertaken employing the 10% change-in-estimate method described by Greenland [24]. In this approach, variables that remain significant after initial adjustment are placed into a second model. Using the backward-deletion algorithms, the second model commenced with all potential variables (including the exposure variable of interest and those that were forced into the model). As each variable is dropped from the second model, those that result in a 10% change in the estimated exposure effect are selected to remain in model, whereas those that do not are deleted. All factors in the multivariable models had less than 10% missing data.

Population attributable risks were calculated using published methods [25]. The interaction between kava use (previous 12 hours) and alcohol use (previous 12 hours) on the risk of four-wheeled motor vehicle crash was tested statistically. Sensitivity analyses investigated the influence of factors that could bias the effect estimates.

## Results

### Participant characteristics and response rates

**Fig 1:**
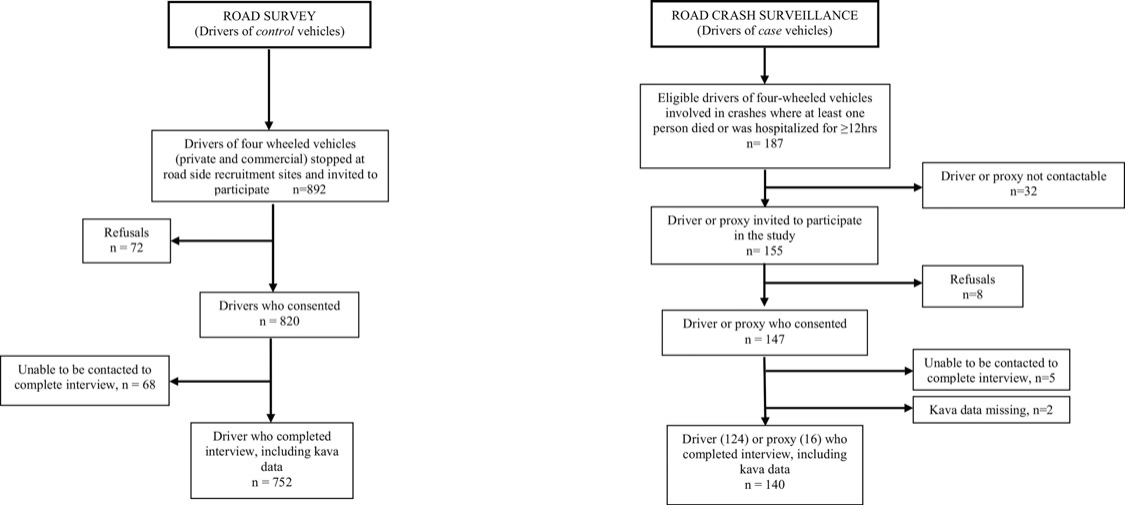
Case and control selection and recruitment, Viti Levu, Fiji, 2006.

Of the 187 eligible case vehicles, 140 (74.9%) drivers or their proxies completed interviews including information on kava consumption (Fig 1). Eight (4.3%) of the eligible drivers declined to participate, and the remainder (20.8%) could not be contacted, were unable to complete the interview or did not provide kava consumption data. Of the 892 control vehicles, 752 (84.3%) drivers completed interviews, 72 (8.1%) declined, 68 (7.6%) were untraceable or could not be interviewed within one month of consenting to participate (Fig 1).

In both cases and control vehicles, over 90% of drivers were male and more than 70% were aged less than 45 years. Most four-wheeled motor vehicles in the study were cars (Table 1).

**Table 1:** Distribution of kava consumption and potential confounders for cases and controls, Fiji, 2006.

Characteristics	No. (%) of Drivers	
	Case (n = 140)	Control (n = 752)^a^
**Age of drivers (years)**		
15–24	19 (13.6)	84 (12.9)
25–34	48 (34.3)	220 (31.8)
35–44	36 (25.7)	223 (29.3)
45+	37 (26.4)	223 (25.9)
Declined to answer/missing data	0 (0)	2 (0.3)
**Gender**		
Female	6 (4.3)	35 (6.8)
Male	134 (95.7)	717 (93.2)
**Ethnicity**		
Fijian (iTaukei)	42 (30.0)	154 (21.9)
Indian	96 (68.6)	551 (70.1)
Other	2 (1.4)	47 (8.0)
**Household Income ($FJD)**		
≥$20,000	29 (20.7)	231 (35.3)
$10,000 - $19,999	38 (27.1)	239 (38.1)
<$10,000	66 (47.1)	275 (25.8)
Declined to answer/missing data	7 (5.0)	7 (0.9)
**Acute Kava use (previous 12 hours)**		
No	108 (77.1)	698 (95.7)
Yes	32 (22.9)	54 (4.3)
**Usual Kava use (past 12 months)**	** **	
None to less than monthly	46 (32.9)	374 (58.3)
Once a month to once a week	50 (35.7)	221 (25.7)
Several times a week to daily	44 (31.4)	157 (16.0)
**Acute alcohol use (previous 12 hours)**		
No	120 (85·7)	734 (96.5)
Yes	20 (14·3)	18 (3.5)
**Time of crash**		
0500 – 1200hrs	42 (30.0)	219 (9.8)
1201 – 1900hrs	50 (35.7)	387 (78.9)
1901 – 0200hrs	48 (34.3)	146 (11.3)
**Vehicle speed (km per hour)**		
≤50	52 (37.1)	502 (74.4)
51–60	29 (20.7)	160 (21.2)
≥61	46 (32.9)	90 (4.5)
Missing data	13 (9.3)	0 (0)
**Car type**		
Cars	88 (62.9)	468 (67.3)
Minibuses/vans	35 (25.0)	257 (29.8)
Trucks	17 (12.1)	27 (2.9)
**Wet road condition**		
No	109 (77.9)	680 (94.0)
Yes	31(22.1)	72 (6.0)
**Type of driving licence**		
Full licence	114 (81.4)	712 (94.2)
Provisional/ no licence	26 (18.6)	40 (5.8)
**Driver in a crash in the past 5 years**		
No	126 (90.0)	620 (79.7)
Yes	13 (9.3)	130 (20.3)
Missing data	1 (0.7)	2 (<0.1)

^a^ weighted proportions.

### Kava consumption, confounding and crash-related injury

**Fig 2:**
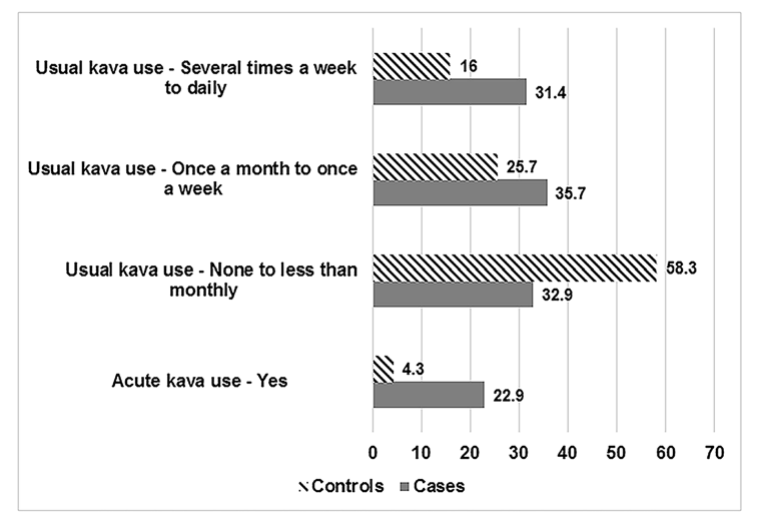
Distribution of acute (previous 12 hours) and usual (past 12 months) kava use among cases and controls.

Among drivers of case and control four-wheeled motor vehicles, 22.9% (32/140) and 4.3% (54/752) respectively, reported drinking kava in the 12 hours prior to the crash or survey (Fig 2). Nearly two times more drivers of case vehicles (31.4%; n = 44/140) reported drinking kava ‘several times a week to daily” in the past 12 months as compared to drivers of control vehicles (16%; n = 157/752).

There was a six-fold excess in the odds of injury-involved road traffic crashes among those consuming kava in the previous 12 hours compared with those who did not, after controlling for the potential socio-demographic confounders of age, gender, ethnicity, and household income (OR: 6.59; 95% CI: 2.88–15.09) (Table 2). Following additional adjustments for usual kava use (past 12 months), acute alcohol use (previous 12 hours), time of crash, vehicle speed and wet road condition, the associated odds ratio remained significant at 4.70 (95% CI: 1.90–11.63) (Table 2). The associated population attributable risk (PAR) was 18.37% (95% CI: 13.77–22.72).

**Table 2:** Association of self-reported kava use with risk of car crash resulting in injury, Fiji, 2006.

Variable	Unadjusted Odd Ratio^a^	*p*-value	Adjusted Odds Ratio^b^	*p*-value	Adjusted Odds Ratio^c^	*p*-value
	(95% CI)		(95% CI)		(95% CI)	
**Acute kava use (previous 12 hours)**						
No	1.00	<0.001	1.00	<0.001	1.00	0.001
Yes	6.57 (2.91, 14.80)		6.59 (2.88, 15.09)		4.70 (1·90, 11.63)	
**Usual kava use (past 12 months)**						
None to less than monthly	1.00	<0.001	1.00	<0.001	1.00	0.04
Once a week to once a month	2.47 (1.45, 4.22)		2.26 (1.27, 4.02)		1.75 (0.96, 3.19)	
Several times a week to daily	3.48 (2.01, 6.04)		3.20 (1.68, 6.07)		2.04 (1.10, 3.79)	

CI: confidence interval

^a^ Adjusted for sampling design

^b^ Derived from a multivariable logistic regression model that was adjusted for age, gender, ethnicity and household income

^c^ Derived from a multivariable logistic regression model that adjusted for age, gender, ethnicity, household income, acute kava use (previous 12 hours), usual kava use (past 12 months), acute alcohol use (previous 12 hours), time of crash, vehicle speed, wet road condition.

We further explored the data to assess potential effect modifiers and the influence of biases. We found no interaction between the main effect of interest (driving following kava use and injury-involved crashes) and the alcohol consumption in the same recall period (p = 0.72). Given the potential for misclassification of exposures, a sensitivity analysis was undertaken where data from the 16 proxy respondents were excluded. There was minimal change in the main effect of interest, driving after kava use (OR: 4.93; 95% CI: 1.97–12.35) or the associated PAR (18.71%; 95% CI: 14.25–22.95).

We also examined the association injury-involved crash risk with kava use over the previous 12 months. Monthly to daily use was reported by 67% and 42% of drivers of case and control four-wheeled motor vehicles, respectively. After adjusting for socio-demographic variables, the odds of injury-involved crashes were significantly higher among those reporting kava drinking ‘once a month to once a week’ (OR: 2.26; 95% CI: 1.27–4.02) and ‘several times a week to daily’ (OR: 3.20; 95% CI: 1.68–6.07) when compared with the reference category of ‘none to less than monthly’. (Table 2) Although these effects were attenuated when the estimates were adjusted for other confounders, the associated odds of crash remained significant for kava drinking ‘several times a week to daily’ (OR: 2.04; 95% CI: 1.10–3.79).

Driving within 12 hours of drinking kava (main effect of interest) and usual kava use (frequency of kava use in the previous 12 months) could be considered as factors in the same causal pathway. When usual kava use was excluded from the main effect model, the odds associated with acute kava use increased to 6.04 (95% CI: 2.38–15.28) with an associated PAR of 19.47% (95% CI: 15.82–22.95).

## Discussion

This case-control study conducted in Fiji (one of several Pacific Island countries where social or recreational use of the substance is common) found a four-fold excess in the odds of crash involvement associated with driving within 12 hours of consuming kava. This finding remained significant after adjustment for confounding factors including self-reported alcohol use. The related population attributable risk suggests that avoiding driving following kava consumption could reduce the population burden of these injuries by up to 18%. Frequent use of kava over the previous 12 months was also associated with increased odds of injury-involved crashes. To our knowledge, this is the first study to investigate the contribution of driving following the use of kava to serious injury-involved MVCs[9].

Our study was not designed to examine the risk posed by therapeutic doses of kava as found in herbal remedies. However, our findings support concerns raised by previous researchers [26, 27] and the media [28, 29] about driving after consuming kava. The Fiji Land Transport Authority has proposed banning drivers of public service vehicles from consuming kava while on work duties [30], revealing increasing support by policymakers to discourage driving following kava use. However, implementing and monitoring this strategy remains challenging in the absence of readily accessible objective measures of recent kava use and inadequate information on the levels of kava that are of greatest concern [31].

The concerns raised by our findings do not discount some therapeutic benefits of kava, nor do these counter the social and cultural significance of this beverage in Pacific communities where it is commonly consumed. However, as with alcohol and substances that have sedative effects, kava could influence crash risks through theoretically plausible biological mechanisms. For example, the GABAergic effects following the ingestion of large doses of kava include impaired performance on tasks requiring high cognitive demand and visual attention [13, 32, 33], and prolonged reaction times in neurocognitive function tests [34, 35] Although experimental studies suggest that pharmacological doses (180–300 mg kavalactones) of kava produce relatively little effect on driving performance [26, 27, 36–38], the doses investigated were substantially lower (50 to 100 times less potent) than those commonly consumed in recreational settings [9].

The extent to which alcohol potentiates crash risk due to kava requires further investigation. A human experiment conducted by Foo and Lemon revealed that kava alone (in doses found in anti-anxiolytic herbal medications) had no effect on the measures of sedation, cognition, motor coordination, intoxication and willingness to drive a car [36]. However, larger negative effects were detected when kava was combined with alcohol, and this effect was greater than the negative effects of alcohol alone. While we did not observe a significant interaction with alcohol in our study (which may relate to the limited statistical power to investigate this interaction), the experimental data suggests the risks of kava could be greater in the context of co-ingestion of alcohol, a practice (“wash down”) that is reasonably common in Fiji [39].

In this population-based study, case ascertainment was likely to be complete given the prospective recruitment strategy implemented at all hospitals and mortuaries receiving trauma patients in the region, minimising recruitment bias. Controls were sampled in proportion to the amount of driving undertaken, in preference to recruiting from an existing licensed driver database or vehicle register which do not account for driving time. However, the findings must be interpreted in light of other limitations. The study estimated the amount of kava consumed in the 12 hours prior to driving using self-reported information from participants. We were unable to standardise the quantities involved due to variations in kava cultivars, concentrations and drinking cup sizes. Objective assessment of kava remains a challenging issue in the Pacific context where laboratory testing is difficult to implement. Further research is required to establish dose-response relationships and patterns of consumption that pose the greatest crash risk. We did not gather information about environmental factors such as roading design, traffic flow, and other road engineering factors that may have played a role in the road traffic crashes in this study [40–42].

While relatively few case records had missing information in key variables relevant to this analysis, proxy reports may have resulted in misclassification of some exposure measures. However, a sensitivity analysis excluding proxy data found the influence on the effect estimate to be minimal. Differential recall of information by cases and controls can result in recall bias [43]. The methods used to minimise recall bias in this study included the standardised administration of identical exposure questions for drivers linked to a specific event (crashes for cases and a roadside stopping site for controls). While we achieved good response rates, those who did not respond may have differed systematically from those who did with respect to relevant exposures. In our multivariable regression models, we adjusted estimates to control for factors known to be associated with poor response, such as age and socio-economic status, as well as other demographic, lifestyle and travel-related variables. However, residual confounding cannot be excluded, a common challenge with observational designs.

In summary, our study found that driving following the recreational consumption of kava was associated with an excess risk in serious-injury involved road crashes in Fiji. While crash risks associated with lower doses of kava as found in some herbal remedies and the dose-response relationships more generally require further investigation, the precautionary principle would suggest that avoiding driving following kava use should be an explicit road safety target, particularly in communities where recreational use is common.

## Supporting Information

S1 Questionnaire (DOCX)

S2 Questionnaire (DOCX)
