# Supplementary material for: Driving following Kava Use and Road Traffic Injuries: A Population-Based Case-Control Study in Fiji (TRIP 14)
Source: PLoS One. 2016 Mar 1;11(3):e0149719. doi: 10.1371/journal.pone.0149719 (PMC4773145; doi:10.1371/journal.pone.0149719)
Supplement: S1 Questionnaire — (DOCX) [file pone.0149719.s001.docx]

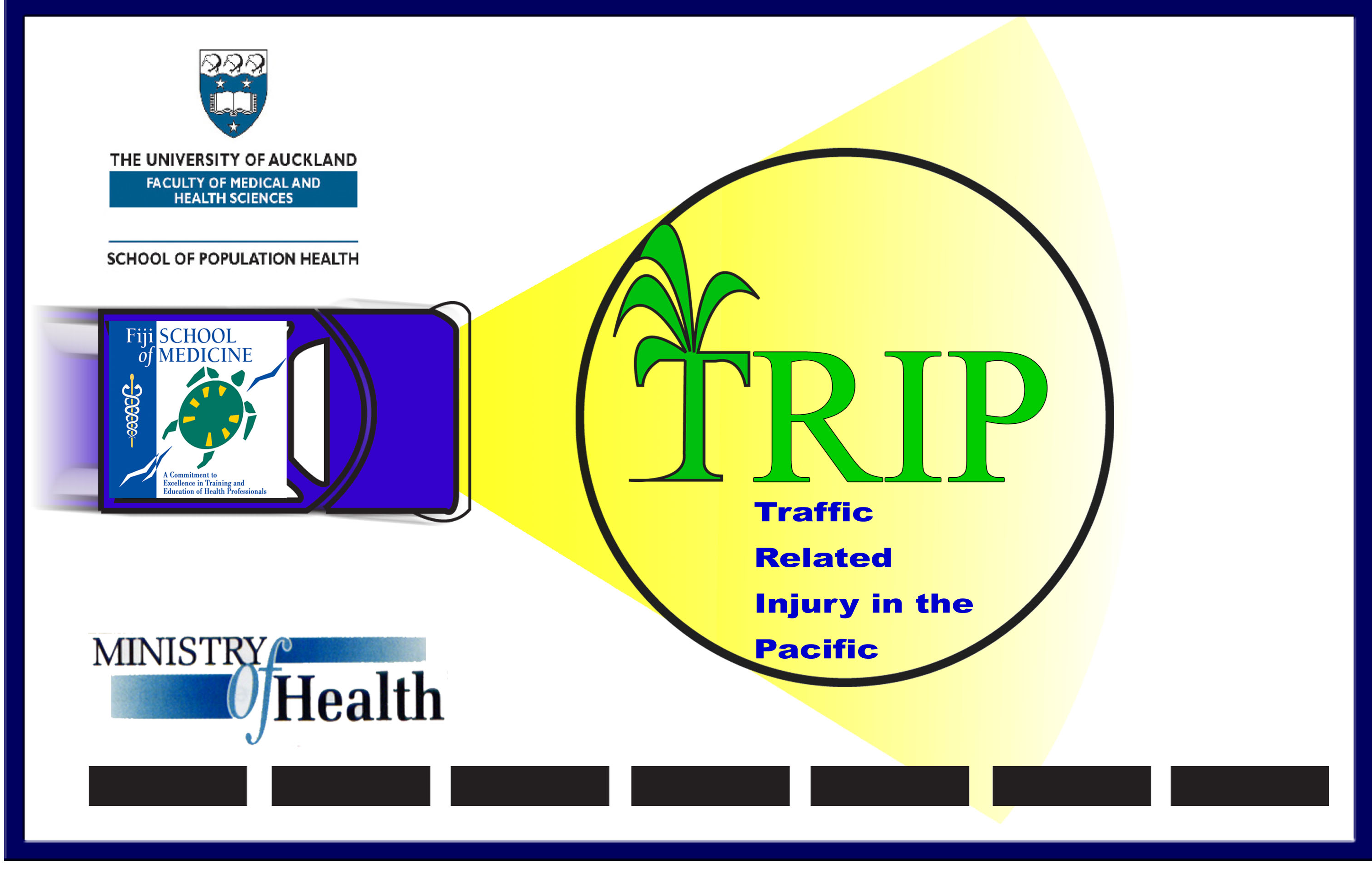


**Traffic Related Injury in the Pacific Project**

Case driver Questionnaire

(English)

Study Number

--

TRIP

Case Driver Questionnaire

 **Researcher code**

 /  /  **Date of Interview** (*dd/mm/yy)*

 1 Telephone **Mode of Interview**

 2 Face to face

 3 Combination

**Proxy Interview?**

 1 No

 2 Yes

**For proxy interview only:**

Reason for use of proxy respondent:

 1 Driver died

 2 Driver seriously injured

 3 Other *please specify* ________________________________

**Was the proxy in the car?**

 1 No

 2 Yes

**Relationship of proxy to driver:**

 1 Parent

 2 Spouse

 3 Other family

 4 Friend

 5 Other *please specify _____________________________*___

Section A

Vehicle Details

We would like to know something about the vehicle you were driving at the time of the crash.

**1. What type is the vehicle?** *(Read all out and tick one box only. You may be able to use the classification on the registration sticker)*

 1 Private car

 2 Taxi

 3 Commercial minibus

 4 Other van/minibus

 5 Pick-up (open “Ute” with tray)

 6 Truck

 7 Other (including rental vehicle) *(please specify)* ____________________________________

**2. For the vehicle, what is the:**

 a) Brand? (*e.g. Nissan*) ____________________________________

 b) Model*? (e.g. Sunny*) ____________________________________

 c) Year? ____________________________________

 d) Colour? *(indicate closest colour from:*

*white, yellow, grey, black, blue, red, green, brown, or silver)____________________________________*

 e) Tone? *(indicate either light or dark)* ____________________________________

**3. What is the vehicle’s registration number (i.e. the number on the number plate)?**  *(Even part of the number is useful)*



*(For questions 4- 7 don’t read out the options)*

**4. At the time of the crash, did the vehicle have a current LTA Fitness Test?**

**5. Has the vehicle ever failed a Fitness Test?**

**6. Has the vehicle tyre pressure been checked in the past 3 months?**

1. **Has the vehicle been modified (e.g. suspension raised or lowered, wide tyres, loud muffler, Mag wheels)?**  *(This implies changes to the structure)*

**Does the vehicle have:**

**8. Bull-bars?**

**9. Airbags?**

**10. Four-wheel Drive?**

**11. Are seatbelts fitted in the vehicle?** *(Tick one box only, read out if hesitant)*

 1 Front and rear

 2 Front only

 3 Rear only

 4 No seatbelts

***Yes No Don’t Know***

(2) (1) (99)

**  **

**  **

**  **

**  **

**  **

**  **

**  **

Section B

Circumstances of crash

Now I would like to ask you some questions about the crash you were involved in.

**12. What was the date and day of week on which the crash occurred?**

**Date:** *(dd/mm/year)* ** /  / **

**Day of week:**

 1 Monday

 2 Tuesday

 3 Wednesday

 4 Thursday

 5 Friday

 6 Saturday

 7 Sunday

**13. What was the time of the crash?**

 :  *(please use 24 hour clock when entering time)*

**14. Where did the crash happen?** (*Please describe the location as precisely as possible, including which side of the road they were traveling on)*

**______________________________________________________________________________________________________________________________________________________________________________________________________________________________**

**15. What was the main reason for this trip?** *(Read out if participant is hesitant)*

 1 Just driving around with no real plan

 2 Going to or from work

 3 Driving as part of your work

 4 Other *(please specify)* ________________________

**15b. How many vehicles were involved in the crash (including your vehicle)?**  *(Include all vehicles whether damaged or not but do not include parked vehicles involved)*

 1 One (e.g. hitting pedestrian, animal, obstacle)

 2 Two

 3 Three or more

**16. Were you wearing your seatbelt at the time of the crash?**

 1 No

 2 Yes

**17. How fast do you think you were traveling immediately before you were aware of the crash?** *(If unsure get best approximation and probe with e.g. were you in a built up or rural area?)*

 km/hr

**18. How long on this trip had you been driving (before the crash), since having a break of one hour or more?**

 hours  mins

1. **In the 24 hours before the crash, how many times did you sleep? When was that?** *(Please use time prompt and 24 hr clock, e.g. “If your crash was at 1000 Tuesday, how many sleeps had you had between then and 1000 Monday?”)*

**Sleep 1**  :  **to**  : 

**Sleep 2**  :  **to**  : 

**Sleep 3**  :  **to**  : 

**20. In the week before the crash, how many nights did you sleep for 7 hours or more?** (“*Nights” means mostly between 11pm and 7am)*

 nights, out of 7

**21. Which of the following best describes your level of alertness in the 15 minutes prior to the crash?**  *(Read out all the options, as many times as necessary)*

 1 Felt active, wide awake

 2 Relaxed and awake but not fully alert

 3 Difficulty staying awake, was beginning to lose track

 4 Felt sleepy, would have preferred to lie down

**22. Had you had any alcohol in the 12 hours before the crash?** *(Use time prompts for all alcohol questions)*

 1 No *If no, go to Q 26*

 2 Yes

**23. What alcohol did you have to drink in the 12 hours before the crash?**  *(So if the crash was at 1000 Tuesday, ask how much they had to drink from 2200 Monday onwards. Always ask “anything else” after each addition to the table)*

|  | Type of drink (e.g., Fiji Gold Beer) | Size of drink (e.g., stubby, nip) | Number of drinks | Office Use only  (Alcohol grams) |
| --- | --- | --- | --- | --- |
|  |  |  |  |  |
|  |  |  |  |  |
|  |  |  |  |  |
|  |  |  |  |  |
|  |  |  |  | **Total:** |

|  | Glass type | ml |  |  | Glass type | ml |
| --- | --- | --- | --- | --- | --- | --- |
| Beer: | *"old fashioned glass" for taki* | *125* |  | Wine: | *white wine glass* | *190* |
|  | *medium glass (high Ball)* | *285* |  |  | *red wine glass* | *190* |
|  | *mug (schooner)* | *285* |  |  | *balloon glass (for port)* | *70* |
|  | *jug (pitcher)* | *1425* |  |  |  |  |
|  | *stubby/can* | *375* |  | Spirits: | *nip* | *30* |
|  | *1/2 pint glass(for draft beers)* | *350* |  |  |  |  |
|  | *pint glass (for draft beers)* | *750* |  | Tequila: | *shot* | *30* |

**24. What alcohol did you have to drink in the 6 hours before the crash?**  *(So if the crash was at 1000 Tuesday, ask how much they had to drink since 0400 Tuesday. Always ask “anything else” after each addition to the table)*

|  | Type of drink (e.g., Fiji Gold Beer) | Size of drink (e.g., stubby, nip) | Number of drinks | Office Use only  (Alcohol grams) |
| --- | --- | --- | --- | --- |
|  |  |  |  |  |
|  |  |  |  |  |
|  |  |  |  |  |
|  |  |  |  |  |
|  |  |  |  | **Total:** |

**25. Had you had any kava in the 12 hours before the crash?** *(May need cueing, prompting*)

 1 No *If no, go to Q 29*

 2 Yes

**26. How much kava did you drink in the 12 hours before the crash?** *(Use prompts and find out:*

*Number and size of bags: _______________ Number of people drinking: _______________*

*How long did you sit for the session and therefore how many of the 12 hours before the crash were you*

*drinking kava? _______________________*

**27. How long before the crash did you stop drinking kava?**

 hrs  mins

**28. Had you used any marijuana in the 6 hours before the crash?**

*(This includes hash, weed, cannabis, grass, pot, “smoking dope” or a “joint”, ganja)*

 1 No

 2 Yes

*(Remind subject that all information is strictly confidential, e.g. it will not be shared with law-enforcement agencies or insurance companies).*

**29. Had you taken any other recreational drugs in the 6 hours before the crash?**

*(If they are confused read out options. This includes speed (amphetamines), datura, LSD (acid), heroin, morphine, methadone, cocaine, ecstasy, amyl nitrite (rush, poppers, ram), solvents (glue, gas, benzene, punpun, plastics), prescription drugs for fun, mushrooms and others)*

 1 No

 2 Yes *(please specify) ____________________________*

**30. Were you using a cell phone or car phone at the time of the crash?**

 1 No

 2 Yes - hand held

 3 Yes - hands free

**31. At the time of the crash were you distracted in any other way, e.g. attending to passengers, lighting cigarette, adjusting radio or CD or windows or air conditioning, noticing something outside the car etc.?**

 1 No

 2 Yes *(please specify) _____________________________*

**32. How many other people were with you in the vehicle?**



*(If minibus, go to Q 34)*

**Can you tell me the age of each of these people, where they were seated, and if a seatbelt or child restraint was available and worn?** (*Driver may need to give best estimates of age)*

|  | Age | Front/back  seat | Seatbelt  available | Seatbelt worn | Child res-traint used | Don’t know |
| --- | --- | --- | --- | --- | --- | --- |
| Person 1 |  |  |  |  |  |  |
| Person 2 |  |  |  |  |  |  |
| Person 3 |  |  |  |  |  |  |
| Person 4 |  |  |  |  |  |  |
| Person 5 |  |  |  |  |  |  |
| Person 6 |  |  |  |  |  |  |
| Person 7 |  |  |  |  |  |  |
| Person 8 |  |  |  |  |  |  |

**At the time of the crash***… (don’t read out options)* ***No Yes***

**33. Was it raining?**  1  2

**34. Were your headlights on?**  1  2

*(Ask even if day-time crash)*

Section C

Personal Factors

I would now like to ask you some questions about your health and lifestyle. You may find that some of the questions are similar to ones asked previously, but I have to ask them all.

**35. Before the crash would you say your health was generally…?**  *(Read out options)*

 1 Excellent

 2 Very good

 3 Good

 4 Fair

 5 Poor

Sleep

**36. Have you ever been told that you regularly snore loudly?**

 1 No

 2 Yes

**37. Have you ever been told that you stop breathing while you’re asleep?**

 1 No

 2 Yes

**38. Have you ever been told that you appear to choke while you’re asleep?**

 1 No

 2 Yes

Now I would like to ask you some questions about drinking alcohol.

**39. Have you ever drunk alcohol one or more times a month?**

 1 No *If no, go to Q 49*

 2 Yes

**40. At present do you drink alcohol one time a month or more?**

 1 No *If no, go to Q 45*

 2 Yes

**41. About how often do you drink alcohol?** *(Please tick one box, read out if participant initially hesitant*)

 1 6-7 days a week

 2 4-5 days a week

 3 2-3 days a week

 4 Once a week

 5 Once every 2 weeks

 6 Once a month

***For the next two questions please refer to the following guide.***

|  | Glass type | ml |  |  | Glass type | ml |
| --- | --- | --- | --- | --- | --- | --- |
| Beer: | *“old fashioned glass” for taki* | *125* |  | Wine: | *white wine glass* | *190* |
|  | *medium glass (high Ball)* | *285* |  |  | *red wine glass* | *190* |
|  | *mug (schooner)* | *285* |  |  | *balloon glass (for port)* | *70* |
|  | *jug (pitcher)* | *1425* |  |  |  |  |
|  | *stubby/can* | *375* |  | Spirits: | *nip* | *30* |
|  | *½ pint glass (for draft beers)* | *350* |  |  |  |  |
|  | *pint glass (for draft beers)* | *750* |  | Tequila: | *shot* | *30* |

**42. On an average day when you drink alcohol how many drinks would you usually have in total?**  *(Always ask “anything else” after each addition to the table)*

|  | Type of drink (e.g., Fiji Gold Beer) | Size of drink (e.g., stubby, nip) | Number of drinks | Office Use only  (Alcohol grams) |
| --- | --- | --- | --- | --- |
|  |  |  |  |  |
|  |  |  |  |  |
|  |  |  |  |  |
|  |  |  |  |  |
|  |  |  |  | **Total:** |

**43. In the PAST 3 MONTHS, what is the largest number of drinks that you had on any one day?** *(Always ask “anything else” after each addition to the table)*

|  | Type of drink (e.g., Fiji Gold Beer) | Size of drink (e.g., stubby, nip) | Number of drinks | Office Use only  (Alcohol grams) |
| --- | --- | --- | --- | --- |
|  |  |  |  |  |
|  |  |  |  |  |
|  |  |  |  |  |
|  |  |  |  |  |
|  |  |  |  | **Total:** |

**44. Have you ever felt you should cut down on your drinking?**

 1 No

 2 Yes

**45. Have people annoyed you by criticizing your drinking?**

 1 No

 2 Yes

**46. Have you felt bad or guilty about your drinking?**

 1 No

 2 Yes

**47. Have you ever had a drink first thing in the morning to steady your nerves or get rid of a hangover?**

 1 No

 2 Yes

**48. Have you used any marijuana during the past 12 months? (This includes hash, cannabis, grass, pot, “smoking dope” or a “joint”, ganja). If yes, how often?**  *(Read out options 2-5 if participant initially hesitant)*

 1 No

 2 Less than once a month

 3 Once a week –once a month

 4 Several times a week

 5 Every day

**49. Have you used any other recreational drugs during the past 12 months?**

*(If they are confused read out options. This includes speed (amphetamines), datura, LSD (acid), heroin, morphine, methadone, cocaine, ecstasy, amyl nitrite (rush, poppers, ram), solvents (glue, gas, benzene, punpun, plastics), prescription drugs for fun, mushrooms and others)*

 1 No

 2 Less than once a month

 3 Once a week - once a month

 4 Several times a week

 5 Every day

**50. Have you had any kava in the past 12 months?** *(Read out options 2-5 if participant initially hesitant)*

 1 No

 2 Less than once a month

 3 Once a week - once a month

 4 Several times a week

 5 Every day

I would now like to ask you some questions about your emotions

**51. During the past 4 weeks have you been bothered by…**

***No Yes***

**a) Feeling down, depressed or hopeless?**  1  2

**If “yes”, do you want help with this?**

 1 No

 2 Yes, but not today

 3 Yes

**b)** **Little interest or pleasure in doing things?**  1  2

**If “yes”, do you want help with this?**

 1 No

 2 Yes, but not today

 3 Yes

**52. During the 12 months before the crash/survey, had you considered taking your own life?**

 1 No

 2 Yes

*(Make notes on any concerns or comments regarding their responses)*

Medications

1. **Had you taken any sleeping tablets during the 24 hours before the crash?**

 1 No

 2 Yes

*If YES, please give details (name of tablets, time taken, dose/number of tablets):*

_________________________________________________________________­­­­­­­­­­­­­­­­­­­­­­­­­­­­­______________________________________________________________________________________________________________________________________________________________________________________________________________________________

**54. At the time of the crash were you taking regular medication for depression or anxiety?**

 1 No

 2 Yes

*If YES, please give details (name of tablets, time taken, dose/number of tablets):*

______________________________________________________________________________________________________________________________________________________________________________________________________________________________

Section D

Driving Experience and Habits

I would now like to know something about the length of time you have been driving and your driving experience.

**55. What type of vehicle licence do you hold at present?** (*Tick the single best option, avoid reading out)*

 1 Never had a vehicle license *Go to Q 61*

 2 Disqualified / suspended

 3 Provisional license *(specify class) ________________________* *Go to Q 61*

 4 Full Fiji vehicle license *(specify class*) _____________________

 5 Overseas license

(Classes: 1: motor cycle; 2: Private, Rental, Goods < 3.5 tonnes; 3: Taxis, Hire ; 4:Carriers 5: bus; 6: goods > 3.5 tonnes; 7:articulated; 8:tractors; 9 Other heavy/Construction vehicles.)

**56. Did you have any professional driving lessons when you were learning to drive?**

 1 No *(go to next question)*

 2 Yes

**If yes, how many?**

 lessons

1. **For how many years have you driven a car or van regularly (i.e. at least 3 times per week)?**  *(If driving hasn’t been continuous, add together periods of regular driving. If less than one year, code 00)*

 years

1. **During an average week before the crash/survey, how many hours would you spend driving a car or van on the road?**  *(Go through usual driving activities day by day and calculate below)*

 1 5 hours or less *Calculation:*

 2 more than 5, less than10 hours

 3 more than 10, less than 20 hours

 4 more than 20, less than 30 hours

 5 More than 30 hours

1. **Since gaining your license, have you had additional driver education, defensive driving, advanced driving courses or advanced driving assessment?**  *(Record as much as possible about any course they have undertaken and remember that service vehicle drivers must have done defensive driving courses)*

 1 No

 2 Yes

*If YES, please specify courses and dates:*

______________________________________________________________________________________________________________________________________________________________________________________________________________________________

**60. Over the past 5 years, have you been the driver in a crash?** *(Include crashes that involved any road vehicle i.e. not only cars. Also include crashes that involved only one vehicle. Do not include this crash)*

 1 No *Go to Q 62*

 2 Yes

**If yes, was anyone so seriously injured in the crash that they died or required medical attention?**

 1 No *Go to Q 62*

 2 Yes

**If yes, how many times in the past 5 years have you had crashes like this?**



1. **Over the past 5 years, how many traffic convictions have you had including speeding tickets and drink –driving convictions but not parking tickets?**

*(Do not include this crash)*



The next questions are about your current driving habits and opinions.

*(Use the cue cards provided or get the participant to write down the options for a phone interview)*

***Very Often Some Hardly Never***

***Often Times Ever***

**62. How often do you:**

a) Drive fast just for the thrill of it?  1  2  3  4 5

b) Take some risks when driving

because it makes driving more fun?  1  2  3  4 5

c) Drive 10km/hr or more over the

speed limit?  1  2  3  4 5

**How often do you:**

d) Speed up if someone is trying to

pass you?  1  2  3  4 5

e) Follow very close behind slower

drivers?  1  2  3  4 5

f) Make rude gestures at other drivers?  1  2  3  4 5

**How often do you:**

g) Honk your horn or flash your lights

in anger at other drivers?  1  2  3  4 5

h) Race for the fun of it?  1  2  3  4 5

i) Drive without wearing a seat belt?  1  2  3  4 5

**How often do you:**

1. Drive while talking or texting on a mobile

phone?  1  2  3  4 5

k) Drive while listening to loud music?  1  2  3  4 5

***Very Often Some Hardly Never***

***Often Times Ever***

l) Drive through a red light?  1  2  3  4 5

m) Overtake on continuous lines or

blind corners?  1  2  3  4 5

Section E

Background Information

I would like to ask you a few details about yourself.

**63. What is your date of birth?** *(dd/mm/yy, or estimate year if date unknown)*

// **____________________________**

**64. Are you:** *(If gender is obvious avoid asking, but may be needed for telephone interviews)*

 1 Male?

 2 Female?

**65. Which best describes your marital status?** *(Read all out and tick only one box)*

 1 Single and not previously married

 2 Married or living with a partner

 3 Separated or divorced

 4 Widowed

**66. What ethnic group do you identify with?** (*Tick only one box, avoid reading out)*

 1 Fijian

 2 Indian

 3 Other *(please specify)* ____________________________

**67. What is the highest level of education that you have received or are currently receiving?**

 1 Primary school (class 1 – form 2)

 2 Secondary school (forms 3-7): 1-3 yrs

 3 Secondary school (forms 3-7): >3 yrs

 4 Technical Institute / Vocational School or similar *(e.g. FIT, Montfort Boystown)*

 5 University

 6 Still a secondary school student

 7 Other *(please specify)* ___________________________________________

**68. Which of the following best describes your main occupation at the time of the crash?**

 1 Work for pay *(please specify)* ___________________________________________

 2 Planting and collecting food for your own consumption

 3 Self-employed *(please specify)* ___________________________________________

 4 Student

 5 Domestic duties

 6 Unemployed

 7 Retired

 8 Other *(please specify)* ______________________________________________________________________________________________________________________________________________________________________________________________________________________________

*Please record role as well as industry. If the job title is uninformative, ask what they spend most of their time doing.*

*For subjects who don’t have a current paid job, record their:*

*-***status** *(e.g. retired) __________________________________________________________________*

*-***previous occupation** *(if any) __________________________________________________________*

*-and* **occupation of your spouse** (*if they have one) _________________________________________*

**69. On average, how many hours per week were you working in paid job(s) at the time of the crash?**

 hours *If zero, go to Q 73*

**70. What was your usual work pattern at the time of the crash?**  *(Read out)*

 1 Daytime – no shifts

 2 Rotating shifts with nights

 3 Rotating shifts without nights

 4 Permanent nights

 5 Other *(please specify)* __________________________________________

**71. Do you start work before 6 in the morning, 5 or more nights per week ?**

 1 No *(Go to Q 73)*

 2 Yes

**Do you finish work after midnight, 5 or more nights per week?**

 1 No

 2 Yes

**72. Was your household’s total income, before tax, above or below $20 000 during the past 12 months?** *(Then ask appropriate categories if participant is cooperative)*

 1 Less than $5,000

 2 $5,000-$9,999

 3 $10,000-$19,999

 4 $20,000-$39,999

 5 $40,000-$79,999

 6 $80,000-$159,999

 7 $160,000 or more

 8 Decline to answer

###### END
